# Integrating sustainability in the design process of urban service robots

**DOI:** 10.3389/frobt.2023.1250697

**Published:** 2023-09-27

**Authors:** Michel Joop van der Schoor, Dietmar Göhlich

**Affiliations:** Methods for Product Development and Mechatronics, Technical University of Berlin, Berlin, Germany

**Keywords:** sustainability, product development, urban service robot, social sustainability, sustainable design, service robot

## Abstract

The concept of sustainability and sustainable development has been well discussed and was subject to many conferences of the EU and UN resulting in agendas, goals, and resolutions. Yet, literature shows that the three dimensions of sustainability (ecological, social, and economic) are unevenly accounted for in the design of mechatronic products. The stated reasons range from a lack or inapplicability of tools for integration into the design process, models for simulation, and impact analyses to necessary changes in policy and social behavior. The influence designers have on the sustainability of a product lies mostly in the early design phases of the development process, such as requirements engineering and concept evaluation. Currently, these concepts emerge mostly from performance-based requirements rather than sustainability impact-based requirements, which are also true for service robots in urban environments. So far, the main focus of research in this innovative and growing product branch lies in performance in perception, navigation, and interaction. This paper sets its focus on integrating all three dimensions of sustainability into the design process. Therefore, we describe the development of an urban service robot supporting municipal waste management in the city of Berlin. It is the set goal for the robot to increase the service and support the employees while reducing emissions. For that, we make use of a product development process (PDP) and its adaptable nature to build a specific development process suited to include the three dimensions of sustainability during the requirements engineering and evaluation activities. Herein, we show how established design methods like the life cycle assessment or life cycle costing can be applied to the development of urban service robots and which aspects are underrepresented. Especially, the social dimension required us to look beyond standardized methods in the field of mechanical engineering. Based on our findings, we introduce a new activity to the development process that we call preliminary social assessment in order to incorporate social aspects in the early design phase.

## 1 Introduction

The lack of awareness about sustainability within the robotics community inhibits more human-centered and interdisciplinary thinking to fully capitalize on the potential of automation (Mai et al., 2022). The prospects of robotics and AI include solving problems in the domains of climate change, mobility, sustainability, healthcare, and skills shortage (Hirsch-Kreinsen and Karačić, 2019). Also, the development and future implementation are supposed to further both social welfare and growth together with productivity in different branches of the economy (BMBF, 2019). This comprises not only automation in the industrial sector but also the growing market for service robots (International Federation of Robotics, 2022). Over the last few years, roboticists have been engaging more with the complexity that is posed by the environment, the tasks, and the interactions required for automating services (Haidegger et al., 2013). This is also reflected in the recent appearance and update of norms with regard to service robots as in 8373:2021-11, 22166:2021-02, and 7000:2021. International standards can help to overcome technical barriers in international commerce (Jacobs et al., 2018) and hence are expected to increase the market growth significantly (ISO, 2021a; Zou et al., 2022). In addition to that, the ongoing development of technology and artificial intelligence (AI) facilitates the emergence of service robots and the research for new applications, ranging from drone logistics (Damoah et al., 2021) to social robots in healthcare (Bradwell et al., 2021).

The potential of robotics and AI has been subject to census among experts during conference workshops and survey processes. Although there was a clear agreement on possible benefits, some experts pointed out practical challenges like long-term adoption, slow diffusion, and profitability, as well as the innovative stage of the majority of such technologies. (Hirsch-Kreinsen and Karačić, 2019; Mai et al., 2022).

Also, a large-scale roll-out can entail negative effects and inhibit the accomplishment of set goals, e.g., sustainability. With regard to the ecological dimension, aspects like energy consumption, recycling, and disposal are crucial and have to be considered with equal importance (Grau Ruiz and O’Brolchain, 2022). Human-induced climate change causes severe and irreversible impacts, threatening the wellbeing of humanity, as stated by the Intergovernmental Panel on Climate Change (IPCC) in early 2022 (Rama et al., 2022). Furthermore, service robots will have an impact on the social environments they are deployed. This includes the ever-present fear of job replacement through automation, increasing inequalities, and possible potential for conflicts arising from the human–robot interactions due to the sharing of public spaces (Vinuesa et al., 2020; Hellerung et al., 2022).

We address this issue and want to raise the attention of designers and engineers to consider ecological and social impacts during the design process and consequently react to them. Especially in the early stages where goals and requirements are set up to create and finally evaluate concepts, designers have the most influence on the outcome. For example, Pigosso (2012) found that 60%–80% of the environmental impact of a product is fixed in the early stages. With the ongoing development process, it becomes costlier and more difficult to make changes in the concept and, hence, affect the impact on the eco-system and its role in society (Grau Ruiz and O’Brolchain, 2022).

In order to complement the design process to encompass impacts on sustainability, several papers identified different obstacles. Isaksson and Eckert (2020) conducted workshops and interviews with experts from product development and engineering sciences. They underlined a clear need for tools, methods, and models to help understand impacts on sustainability and the need for support for a transition toward sustainability impact-based requirements. Watz and Hallstedt (2022) argue that despite a plethora of tools and methods for sustainability in product development, the application is still low. For Grau Ruiz and O’Brolchain, (2022), the existing design frameworks and guidelines are not focused on the issues at hand. Another problem resides in the idea of quantifying and monetizing sustainability to evaluate and balance the trade-offs between the economic, ecological, and social dimensions. These are not intuitively resolvable and make such a sustainable design hard to execute (Mattson et al., 2019). Wever and Vogtländer (2020) report complaints from designers that design for sustainability is too complex and labor-intensive, therefore not fitting into their design practices. They express the wish for simple, yet omniscient tools to solve specific problems in an agreeable manner.

From all three dimensions, the social aspect of sustainability has been mostly neglected in the field of product development and in the sustainability agenda itself (Mckenzie, 2004; Mattson et al., 2019). The presented methodology is to the best of our knowledge one of the first attempts to account for all three dimensions of sustainability (ecological, economic, and social) in the product development process (PDP). Since this aspect has so far only been insufficiently integrated into the PDP, we focus on the social dimension. We present a general methodology and practical application in the framework of developing and testing a service robot.

We start by introducing some core definitions to put our use case into a frame of reference in Section 2. Further, we describe the concept of sustainability that we base our work on. Here, we also establish an understanding of the design methods used for the development of our project. Following in Section 3, we describe the method used during the different stages of the early design process of an urban service robot and establish a process model suited for such use cases. The results will be presented in Section 4, going into detail about the impacts we identified during our case study. In Section 5, we discuss the results, their validity, and the possible transferability to other robotics or engineering domains. We conclude with a summary and the implications the method had on the development process in Section 6.

## 2 Related work

### 2.1 Distinction between urban service robots

To clarify the subject, we will introduce definitions from the relevant literature to understand the term “urban service robot”. It consists of two generic terms to be further explicated. First, we clarify the type of robot we relate to, and second, the operational environment is specified.

A robot is understood as a “programmed actuated mechanism with a degree of autonomy to perform locomotion, manipulation, or positioning” (ISO, 2021b). The herein-mentioned term autonomy describes the “ability to perform intended tasks based on the current state and sensing, without human intervention” (ISO, 2021b). The norm differentiates between industrial, service, and medical robots. While industrial robots are defined as operating in an industrial environment, service robots perform tasks for humans or equipment. They are split into personal use, which includes non-commercial tasks and is usually done by laypersons, and professional use, which includes managing commercial tasks that require a trained operator (ISO, 2021b; International Federation of Robotics, 2022). Professional tasks include “inspection, surveillance, handling of items, person transportation, providing guidance or information, cooking and food handling, and cleaning” (ISO, 2021b).

Cities or urban places are described as having “a reasonably large and permanent concentration of people within a limited territory as the common characteristic” (Kuper and Kuper, 2003). Tiddi et al. (2020) emphasize three factors that are essential for such urban spaces, namely, the population, their relationships, and the existing facilities. Facilities can be understood as places frequented by the population for economic or social/cultural matters (Mumford, 1937).

Based on these definitions, we constitute urban service robots (USR) as professional service robots that are deployed in urban spaces and depending on their level of autonomy are operated by trained personnel. The higher the autonomy, the more the USR becomes a co-worker rather than a tool that is used or operated by someone. This distinguishes a USR from a common product during the use phase.

### 2.2 Sustainability

The term “sustainability” emerged from the domain of silviculture and was first brought up in 1713 by a German forester who described the importance of regulating the felling of trees, so it would not exceed the rate of the forest’s regeneration capabilities in order to keep a constant yield without destroying its source (Carlowitz, 1713).

The term was coined on a more general level by the World Commission on Environment and Development (WCED) in their report “Our Common Future” from 1987. They defined sustainability as “development that meets the needs of the present without compromising the ability of future generations to meet their own needs” (Brundtland et al., 1987). In creating this commission in the first place, the UN General Assembly reflected on the growing concerns about environmental problems on a global scale and its possible consequences for economic and social development. Although this is the most commonly referenced definition, it has undergone various interpretations so there is no generally accepted understanding of it (Thomsen, 2013). The long-term goals, referred to as sustainability, and the ways to achieve them, referred to as sustainable development, give room to interpretations, which may differ greatly depending on the conception (e.g., theory of weak or strong sustainability) (Jacobs, 2004). However, most of them share the idea that it requires the reconciliation of an ecological, social, and economic dimension.

#### 2.2.1 Dimensions

In 1992, the United Nations Conference on Environment and Development (UNCED) created Agenda 21 as a comprehensive plan of action to counteract negative impacts induced by humans and thereby gave a first summary of concepts for sustainable development. Again, these pertain to the three elements: ecological, social, and economic sustainability (Wolfgang, 2018). To understand what these dimensions comprise, they will shortly be introduced.

The ecological dimension refers to our natural environment. The primary goals defined in Agenda 21 are the reduction of the usage of nonrenewable resources, the preservation of nature’s capacity for regeneration and ecological values, and a general improvement of its condition (United Nations, 1993). A more recent and detailed reflection of the aspects of the ecological dimension and its current status can be found in the planetary boundaries, which clearly depict the need to reduce environmental impacts (Steffen et al., 2015). To cater toward more ecological sustainability, it is necessary to reduce products’ energy and material consumption, stop the use of hazardous substances, and increase recycling and reuse rates (Thomsen, 2013).

Social sustainability relates “to the wellbeing and quality of life of the society and individuals in current and future generations.” (Thomsen, 2013). In Agenda 21, this was expressed in several statements about reducing disparities, eradicating poverty, and inclusion and strengthening of important groups (Grunwald and Kopfmüller, 2006; Wolfgang, 2018). On a product level, this refers to the impact it has during its whole life cycle. This means ensuring that no human right is violated during any process, but rather increasing the wellbeing and quality of life of involved individuals. This also includes equity in terms of an evenly distributed profit among the people involved (Thomsen, 2013). Besides those factors, Griessler and Littig (2005) and Cocklin and Alston (2003) highlight work and education, social cohesion, institutions, and infrastructure as important conditions in their definition of social sustainability.

The economic dimension is defined as a concept of economic growth that in the long term does not destroy the resources it is based on (Jeronen, 2013). In terms of product development, the economic aspect is often characterized as profitability and contrary to the other two dimensions has always been a part of prevalent design methods (Mattson et al., 2019). Thomsen (2013) and Nowak (2018) add the social aspect and consider the improvement of the quality of life and the produced goods and services to be part of the concept. It is not only about maximizing profits but also a strategy for efficient business models to sustain long-term persistence and support social welfare (Unternehmerlexikon, 2023).

#### 2.2.2 Sustainability models

**FIGURE 1:**
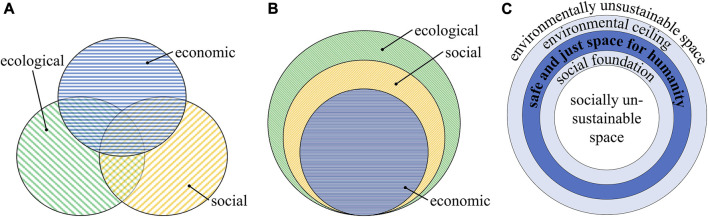
Overlapping **(A)** and interlacing **(B)** circles were adapted from Mckenzie (2004). Donut-shaped model of a sustainable space for humanity **(C)** adapted from Raworth (2012).

The descriptions above comprise a certain overlap of the dimensions. Among several variations of triangles, pillars, or circles, Figures 1A, B show two established illustrations depicting these interdependencies. The three overlapping circles (Figure 1A) represent all dimensions equally, whereas the interlaced circles (Figure 1B) symbolize the dependency of the three dimensions (Mckenzie, 2004). Huckle (2005) describes the overlapping circles (Figure 1A) as the reformist view on sustainable development, balancing economic growth with the social and environmental aspects. He argues that they lack the display of ecological limits in which society and economy can develop healthily and sustainably. In a similar way, Meyer-Abich (2001) argues for the interlacing circles (Figure 1B), denying equity of the dimensions since he sees them as dependent on each other. There would be no society without the natural environment and resources to sustain it and no economy without natural resources and the people to create it (Meyer-Abich, 2001). Another critique of the overlapping circles by Mebratu (1998) is concerned with the possibility of treating them as independent systems since they each have a space with no relationship to one of the others. This is also framed as an area of contradiction, while there is only one small desired zone of interaction to find sustainability (Mebratu, 1998). A rather new model created by Raworth (2012) describes a safe and just space for humanity as a donut-shaped form shown in Figure 1C. This space is confined by an environmental ceiling and a social foundation, which shall not be exceeded. It combines the social dimension as 12 aspects in the middle and the ecological dimension as the nine planetary boundaries on the outside. It proclaims the given boundaries as a starting point for assessing the economy’s activities and wants to utilize the economy to steer into the safe and just space in between (Raworth, 2012).

In 2015, the united nations presented the 2030 agenda that introduced 17 sustainable development goals (United Nations, 2015). A year later, these goals were put into the so-called wedding cake, which sorted the goals into the three categories of biosphere, society, and economy (Stockholm Resilience Center, 2016). The order of these categories reflects the idea of interlacing circles, setting the biosphere as the foundation of society and society as the basis for the economy.

In the same manner as the definition of sustainability, these models differ in meaning and interpretation. Carnau (2011) and Sutton (2004) express that they would rather neglect an exact definition of the concept and concentrate more on identifying what is to be sustained and in which timeframe this is to be included in sustainability policies. Norton (2007) argues that principle stances do not matter as much as the need for applicable tools in order to agree on meaningful actions and implement concrete policy measures. On the contrary, we think it is important to have a common, underlying definition of sustainability and its corresponding model of dependency to create applicable tools. For instance, this is necessary as a reference for prioritization and decision-making during the design process. We follow the interlaced circles (Figure 1B) and acknowledge the dependency and limits of the dimensions.

### 2.3 Sustainability in the design process

The design process is part of the product life cycle and describes the development of a concept from an idea or assignment into a product ready for serial production (2221:2019-11). Autonomous service robots are highly complex mechatronic systems (Haidegger et al., 2013), and managing this degree of complexity in the development of a product requires a structured procedure. Therefore, the revised 2221:2019-11 divides the design process into several activities that will be run through during the process. It is important to note that these activities are overlapping and will be reiterated during the design phases. Each activity yields a certain result, catering toward the next activity or prompting a revision of a step before it ends in final product documentation. As stated in the introduction, we will center this paper around the early stages of design, meaning the first iterations of this process, including prototyping. During these stages, we focus on the “clarification of problem or task” and the “assessment and selection of solution concepts”. In between these steps, the product’s functions and its structure have to be determined for the creation of concepts suitable for a solution to the initial problem. Methods and tools to successfully complete these design steps are well documented in Gericke et al. (2021) and will not be touched upon in further detail throughout this paper.

We make this distinction because we deem the objectives and requirements as the basis for a product’s sustainability. They will shape the elaboration of concepts and hence codetermine the final solution. Secondly, the outcome of the selection process is particularly dependent on the applied set of criteria and their prioritization, which have to be adapted to favor sustainable properties and reach the desired result. The application of methods for assessment is not only suited to select among concepts but also to give directions and support decision-making during the concept phase (Wartzack, 2021).

#### 2.3.1 Product development process models

Models for the PDP support designers in planning and documenting projects, supply suitable tools for finding solutions and decision-making, or provide a basis for higher education in design (Göhlich et al., 2021). Among various models such as French (1999) or Andreasen and Hein (2000), we base our approach on the German norm 2221:2019-11 that cumulated significant and relevant PDP literature to present a general overarching PDP model. The process starts with a set of objectives generated from an internal or external design request, task, or problem. The first activity is a clarification in order to gather the requirements. Therefore, the literature offers methods such as checklists and stakeholder analysis (Bender and Gericke, 2021a), scenario technique (Gausemeier et al., 1998), or the analysis of the product’s life cycle (Roth, 2014). Their goal is to capture a near-complete list of requirements necessary to define the product’s form and functionality and set the scope of all processes around the product, including project management goals and finances. This list serves as a tool for communication accessible to all involved departments and as a measure of the success of the design progress (Bender and Gericke, 2021a).

After creating a number of basic solution concepts, they have to be assessed and rated. The general approach is to derive a set of criteria dependent on the requirements list and rate each solution concept by costs and benefits to reach some kind of a comparable score (2221:2019-11). High-complexity solution concepts and the need for a robust result increase the time spent on this procedure and demand for sophisticated assessment tools such as the utility value analysis or the VDI2225 (Wartzack, 2021). The utility value analysis considers the specified criteria and transforms their values into a unit-less score for comparison. The VDI2225 is a two-dimensional assessment taking technical and economic aspects into account keeping their scores separate for evaluation. Tools like total cost of ownership (TCO) or life cycle costing (LCC) support the economic assessment (Kauf, 2021).

Inherent to all these methods is the technical and commercial point of view as described in 2225 Blatt 3:1998-11 or in the 2221:2019-11 that declares quality, cost, and time as the main objectives. This means that the economic dimension characterized as profitability is already a part of product development (Mattson et al., 2019). The sole factors concerned with the other dimensions of sustainability are mostly tied to the ecological aspect motivated by legal standards and commercial implications (Verein Deutscher Ingenieure, 2002; Verein Deutscher Ingenieure, 2021).

**FIGURE 2:**
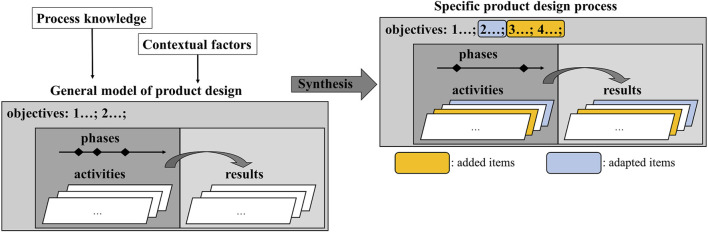
Specific PDP from contextual factors and process knowledge adapted from (2221:2019-11).

However, the VDI2221 provides instructions for the synthesis of a specific PDP by taking contextual factors and process knowledge into account (see Figure 2). This can result in adding or adapting objectives, activities, and their results for one or more specified phases of the PDP. This will be the starting point for our method described in sect. 3.

#### 2.3.2 Design methods for sustainability

Brezet and van Hemel (1997) developed the eco-design checklist and strategy wheel for the United Nations Environment Program (UNEP), which describes possible problems arising in each life cycle stage and poses questions concerning social needs. It features eight eco-design strategies: new concept development, selection of low-impact materials, reduction of materials usage, optimization of production techniques, optimization of the distribution system, reduction of impact during use, optimization of initial lifetime, and optimization of end-of-life system. Besides the first strategy, they all relate to different life cycle stages (van Boeijen et al., 2020). The new concept development strategy targets the idea and objectives of the product and is supposed to encourage a reconsideration of the product’s concept for improvement. Intended for evaluation, it can also be used to complete the list of requirements since the strategies address ecological impact factors.

In the same way, the life cycle assessment (LCA) specified in the DIN EN ISO (2021) can help to add to the list of requirements. The LCA aims to accumulate and evaluate the data of a product’s environmental impact linked to its life cycle stages. In the first step, the system’s scope has to be defined in order to know what has to be included in the life cycle inventory analysis, which constitutes the second step. Here, every process in the product’s life cycle stages is determined and coupled with its inherent energy and material flow. These can be measured, calculated, estimated, or taken from literature. Next is the selection of the impact assessment method, which defines the impact categories, evaluation method, and presentation of results. The most recent and updated method is called the ReCiPe model (European Commission, 2021) and translates the data of step two into environmental impact scores. These can be either taken at the so-called midpoint or endpoint. The first comprises 18 factors and the latter summarizes them into the three factors: Damage to human health, damage to ecosystem, and damage to resource availability. Lastly, the results have to be evaluated by the chosen scope and the validity of the data to derive an assessment of the product and make suggestions for improvement (DIN EN ISO, 2021).

Both the LCA and the eco-design help to assess a product’s ecological impact on the environment. This can be used to derive requirements and rate solution concepts. For social sustainability, there is the social life cycle assessment (S-LCA) operating in the same manner as the LCA and cycling through the four stages: Goal and scope, social life cycle inventory, social life cycle impact assessment, and interpretation (UNEP, 2020). Since our case study deals with the introduction of an autonomous service robot, the use phase differs from other products. It will not be used as a tool but rather put in place to operate on its own or as a co-worker, which makes people on the streets a group of directly affected stakeholders. In order to anticipate possible social implications, we apply complementary methods that the S-LCA does not offer: The social sustainability guideline for automation initiatives (Kohl et al., 2020) and an adaption of the methodology used by Glatte et al. (2019). The social sustainability guideline enumerates 10 indicators of social sustainability divided into four areas: accessibility and equal opportunity, peripheral effects, quantitative effects on work, and qualitative effects on work. It helps to rate each indicator for the applied product or project that results in a proposition of what to do accordingly. The method by Glatte et al. (2019) is a form of participatory design (PD), where stakeholders are invited to workshops. There, they are presented with future scenarios of technical and societal dimensions and made to experience prototypes of so-called speculative design. This way, the designers can learn about desirable and undesirable concepts from the participants and collect additional requirements that did not come up in the first iteration. This does not only further social acceptance but can be a key factor for successful design, revealing stakeholder’s intuitive and intangible expertise (Šabanović et al., 2014; Winkle et al., 2021). Another way of gathering information and including stakeholders is semi-structured interviews (Witzel, 2000) or surveys, which also help quantify the collected data (Bryman, 2012). Seibt et al. (2018) also underline the importance of PD as a means to track social implications throughout the development. More tools and techniques of PD can be found in Brandt et al. (2013).

## 3 Methodology

**FIGURE 3:**
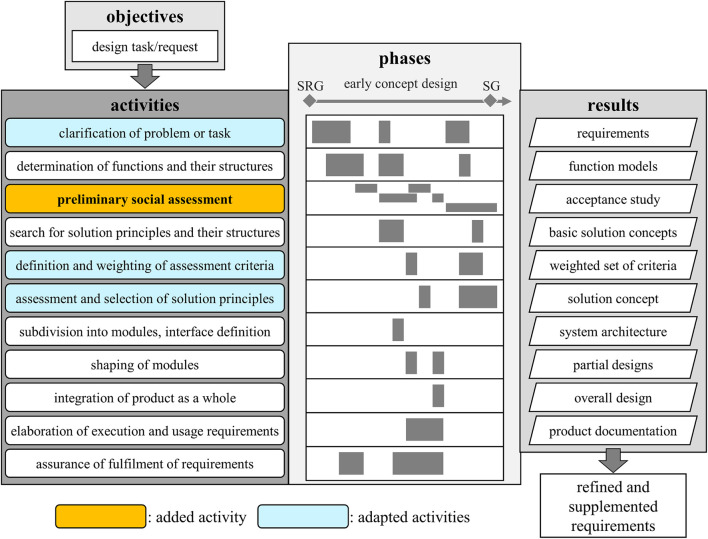
The specific development process for the early concept design phase of our case study with the added and adapted items integrated in accordance with (2221:2019-11).

We suggest a methodology to incorporate a holistic view of sustainability in an early design phase, illustrated in Figure 3. Our approach contains a preliminary assessment process and complements the design steps that were mentioned in Section 2.3. This section provides a thorough description and a step-by-step guideline for our specific PDP created along the lines of the VDI2221 synthesis to match service robots, adding social and ecological aspects. This contains methods and tools that will be explicated in detail in the subsequent section. Furthermore, over the course of this design process, we identify and describe three major conflicts of interest with regard to the added content. Within the methodology, we suggest existing tools to deal with such conflicts. However, there is no final solution within the methodology since this depends very much on the values and strategy of the company or designers, which cannot be provided.

### 3.1 The sequence of design steps

Including social sustainability in the first activity (clarification of problem or task) is complex, especially with the product being a service robot operating in public spaces. The list of stakeholders has to be extended by including the people who frequent those spaces. They neither buy the product nor work with it but, nonetheless, have to share their space and are hence involved and an inherent part of its system or environment.

Therefore, we look at the early concept design phase and introduce an activity that we call preliminary social assessment (PSA). This process focuses on social sustainability and only includes the other dimensions when closely connected to social aspects (e.g., social acceptance can relate to demands for eco-friendly products or reflect on possible market potential). The main outcome, however, will provide social requirements with a focus on the service robot’s use phase. To complete social requirements connected to the remaining life cycle phases, the S-LCA provides key factors in that regard. Exemplary findings from the PSA and aspects from the S-LCA will be collected and converted into a checklist to support designers in this activity (see Table 4).

As seen in Figure 3, we create a phase diagram for a more detailed view. For our context specifically, we want to highlight two additional gates, one at the start and one at the end. The one in front is named the social responsibility gate (SRG) and the one at the end is the sustainability gate (SG). The SRG is derived from the concept of corporate social responsibility and functions as a first check-up of the incoming request or task to be aligned with the company’s own values. The SG concludes the development phase and determines how to proceed based on the gathered results. Both will be described further in the following Section 3.2.1.

For the collection of requirements within the ecological dimension, we provide another checklist generated from the LCA and Eco-Design categories to reduce the impacts of resource depletion, GHG emissions, and other damages to our eco-system. Concerning the economic dimension, we already showed this to be predominant in all relevant literature and norms (Section 2.2.1 and Section 2.3.1) and thus will not add to this subject. The aforementioned lists will complement the “clarification of problem or task” and add social and ecological next to the existent economic and functional aspects.

All four dimensions are crucial in forming a sustainable product. Some requirements can be related or dependent, so optimizing one of them might worsen the performance of another. The different solution concepts created during the design process reflect the impossible task of fulfilling all requirements optimally and vary in their pros and cons. Deciding on a solution with a rather equal distribution among the dimensions or leaning toward one of them is conducted in the assessment, evaluation, and selection activity. Originally, this is described in the activity “assessment and selection of solution concepts”, which in our opinion does not reflect the amount and variety of tasks and results that it contains so we suggest splitting it in two (see Figure 3). The first part contains defining and weighting the assessment criteria, whereas the second part holds the evaluation and selection of a concept. In both parts, we again integrate a social and ecological point of view to keep up with the four dimensions throughout the procedure.

### 3.2 Activities during the early concept design phase

The process chart in Figure 3 illustrates our suggestion of the activities’ sequence during the early concept design phase. For us, this phase signifies an extensive clarification of requirements and an assessment through first concept drafts and prototypes with a focus on all dimensions of sustainability, as opposed to the regular objectives orbiting around time and cost. We begin this section by describing the gates that we placed around the development phase, then explain the PSA and clarify the changes made to the existing activities at the end.

#### 3.2.1 The gates

The social responsibility gate (SRG) marks the beginning of our development process. We want this step to be a quick way to categorize the design request by a number of objectives that reflect the values and corporate strategy of the company or designer. This requires the objectives defining the SRG to be rather easily revisable. For example, the objective to develop a profitable product is paramount to any company or designer but cannot be verified quickly without a first concept or design draft and further investigation. In other words, the objectives have to be formulated in a way that the information stored within the design request can deliver a “satisfying” answer. Since this is susceptible to misunderstandings due to a lack of information or ambiguity, we do not claim the SRG to deliver a final statement, but rather to be a screening and preselection of the design request or task. Uncertainties have to be investigated during the early concept design phase and evaluated at the end marked by the sustainability gate (SG). Although this might be an obvious and standard procedure, we specifically mention this to underline the responsibility that each individual in the line of a development process has and can enact. We make a proposal for a number of objectives from across all dimensions that we came across during our case study in Table 1. It aims to give examples and clarify the concept behind this gate; the list is by no means complete. Additionally, we assign the sustainability dimensions and a short description to each proposal.

**TABLE 1:** Proposal for value-driven objectives to screen any design request or tasks.

Objectives	Description	Dimension(s)
No military purposes, only civil applications	Concerning the initial purpose, ambiguity from unintended or unforeseen repurposing is possible	Social
No surveillance and/or collection of sensitive data	No transgression of privacy (e.g., in support of law enforcement)	Social
Promote diversity, equity, and inclusion	Intended to improve the situation of marginalized and discriminated groups	Social
Benefits a community rather than a single individual	Purposed to economically or socially benefit a larger group of people involved in its life cycle	Social and economic
No loss of jobs	Either a strict policy of not replacing humans at all or coupled with a concept of providing alternative jobs for replaced workers	Social and economic
Longevity and high recycling rate	Intended to be used and deployed as long as possible (no single use), with a high potential for circular economy by modularity or easy dismantling	Ecological and economic
Reduction of carbon footprint	Overall clarity of decreasing ecological impact by improving an existent process and increasing energy efficiency	Ecological and economic
No use of fossil fuels (during the use phase or entire life cycle)	Only use of electrical energy, coupled with a concept of sustainable energy composition	Ecological
No use of toxic materials	No materials with the risk of harming the natural environment and human health to be averted or reversed only at a high cost	All
No destructive purpose	No support or activity with the risk of harming the natural environment and human health to be averted or reversed only at a high cost	All
Application to support one or more goals described in the SDGs	Dedication to improve categories specified in the 17 SDGs by the UN	All

The SG functions as a standard gate, which is common to each development process. The name originates from the special focus on the sustainability evaluation criteria that we establish by the requirements gathered. Over the course of the many activities considered in the process, the goal is to compile a considerable set of data on the project with a sufficient amount of information to base a decision on. How this decision is executed should again be aligned with the values and the corporate strategy of the company or designer. As this is a complex procedure, we act on the assumption that there is more than one individual involved, making the definition and execution of both gates a possible conflict of interest. Since all dimensions are involved, aspects of a qualitative nature are included that can withhold accurate validity, and a decision cannot only be based on numbers. Going about such conflicts requires dialogue and exchanging arguments to try and reach accords even though these might only lead to partial solutions. The approach might be affected and possibly limited by the hierarchy and structure within the company or group of designers.

#### 3.2.2 Preliminary social assessment

**FIGURE 4:**
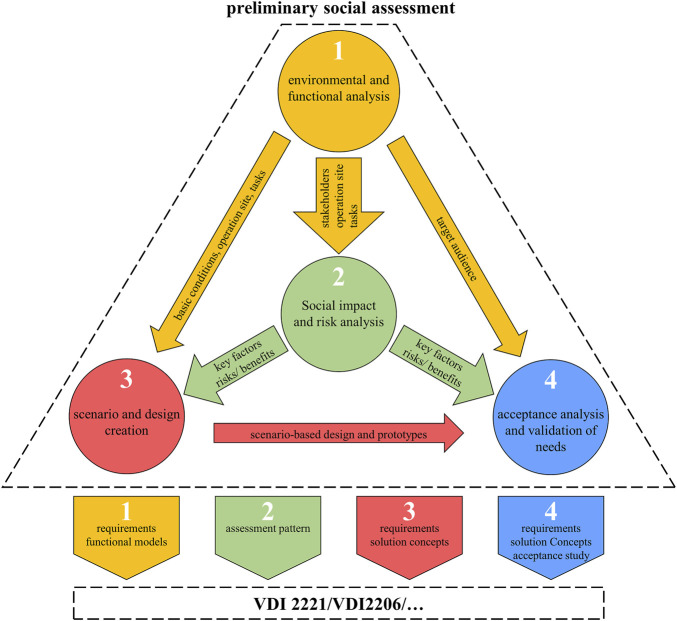
Preliminary social assessment as a separate tool to be integrated into arbitrary development procedures.

Since we introduce the PSA as an addition to the VDI2221, we will explain this separately as a “closed” process, which is illustrated in Figure 4. Presenting it as such enables the PSA to stand for itself and be transferred and integrated into any other PDP like the VDI 2206 to integrate the social dimension. The graph in Figure 4 shows the first step as the environmental (in the sense of surroundings) and functional analysis, which is a combination of the first two activities of the general PDP in the VDI2221. The following three steps of the PSA account for the three separate sequences as seen in the phase chart of Figure 3: the social impact and risk analysis (SIRA), the scenario and design creation, and finally the acceptance analysis and validation of needs. All four steps are intended to provide important information, mostly for the social and technical dimension, to be used in any further design steps of the development methodology (e.g., VDI2221, VDI2206, etc.). In the following, we explain each step and clarify the interaction between them before we address the complementary changes to the other activities from the VDI2221.

**FIGURE 5:**
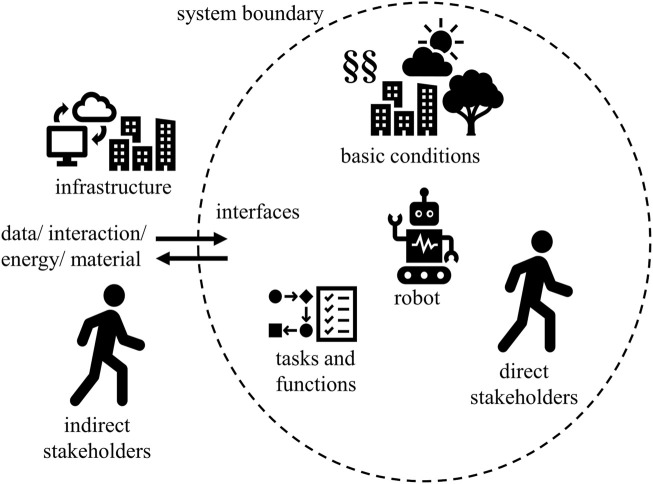
Analysis of the robot’s surroundings and functions to gather information on stakeholders, basic conditions, operation site, and tasks.

The first step necessary in the PSA is the analysis of the robot’s environment and function, illustrated in Figure 5. The goal herein is to clarify what the robot is doing, where it is doing that, with whom, and how. That will result in a set of information on the basic conditions, tasks, and functions of the robot and stakeholders directly or indirectly involved with it. These make up the robot’s system confined by the system boundaries. Additionally, interfaces for communication, energy, or material flow have to be considered in the gathering of information and planning of functions.

In order to implement this step, there is a plethora of tools provided by the product development community (Bender and Gericke, 2021b) (also see Section 2.3.1). Important to note is that after step one, the designers should have a substantial concept and idea of the system surrounding the robot and the challenges it imposes on the fulfillment of its service or task. This aggregation of information serves as a basis for all three following steps and is therefore to be conducted thoroughly.

The second step in the PSA consists of a method that was created on the basis of the S-LCA and is adapted to the assessment of the use phase of automation initiatives (Kohl et al., 2020). The social impact and risk analysis (SIRA) aims to assess technological impacts on its social environment and carve out possible risks that emanate from the proposed robotic application. Investigating this area of social sustainability and getting a better understanding of both negative and positive effects will help shape and influence the outcome of the PDP. The difference to an S-LCA is the timing of our assessment. It does not evaluate an existing product by listing the occurred impacts, but it is taken into the early design phase to estimate impacts and risks to actually avert them for the final product.

Therefore, the basis from step one will provide the designers in this activity with information on the stakeholders involved, the operation site, and the intended tasks. With a first outline of the robot and its surrounding system, the list of 10 factors from four social key issues shown in Table 2 has to be applied. Depending on the project, not all factors may be relevant so the first step aims to identify all factors from the list that will be affected. Kohl et al. (2020) provide a guideline to support the assessment process. Herein, four degrees of applicability from “not needed” to “applicable and urgent” are given to sort those relevant factors. The outcome can be based on the design team’s evaluation and possibly include experts to finalize the first assessment. This can, for instance, be documented in a radar plot as suggested by Kohl et al. (2020) to communicate the key statements. The important output of this step is to sensitize the involved designers and consider the given areas and factors of social sustainability. The relevant key factors and the assessment will transfer toward the two following steps of the PSA to be further considered during any creational and conceptual work.

**TABLE 2:** Social factors to consider for the implementation of a robot (Kohl et al., 2020).

Indicators of social sustainability	
Quantitative effects on work	Loss of employment
	Repositioning
Qualitative effects on work	Monotonous/mentally straining tasks
	Dangerous/physically straining tasks
	Losses in position and task quality
	Polarization of qualification levels
Peripheral effects	Ethical complications/responsibility issues
	Loss of socially valuable services/contacts
	Creation of hostile environments
Accessibility and equal opportunity	Decreasing accessibility and equal opportunity

Step three follows the suggestion of Glatte et al. (2019), which is based on the scenario technique and the so-called speculative design. Bender and Gericke (2021a) also emphasize the creation of product artifacts to explore user experience and evaluation through prototyping. This is necessary to counteract the known mechanisms of the Collingridge dilemma during the development process (Collingridge, 1982). It describes the opposing progression of influence possibilities on the one hand and stakeholders’ engagement and interest on the other. In the early design phase, the possible influence by stakeholders is high, but the engagement and interest are low due to a lack of tangible results or illustrative design studies. As the product becomes more concrete and takes form, the stakeholder’s interest in engaging rises, but the possibility of influence declines (Glatte et al., 2019). Therefore, the gathered information from the first two steps supports designers in the creation of a representation of possible product concepts in their future surroundings to engage stakeholders. While this is done in an early phase of the concept’s elaboration, the possibilities to influence the final outcome are still high.

Creating the scenarios is one part of the three steps of the PSA. Therefore, we follow the description of the scenario technique by Gausemeier et al. (1998). Each scenario will hold implications for the project and deliver a new point of view on the basic conditions that might prevail at the actual time of market entrance. The next part comprises the so-called speculative design (Dunne and Raby, 2014) and prototyping. This means the shaping of first product concepts into physical prototypes. These prototypes do not need to be fully functional but rather demonstrate the intended purpose of showcasing possible outcomes to an audience. This can be described as a translation of the ideas and concepts the designers have of the robot project into an understandable, perceivable object. To successfully do so, the expertise and support of product designers should be considered.

The last step in the PSA embodies the actual engagement of the stakeholders in the design process by conducting surveys, interviews, and workshops. As mentioned before, this is especially important to find out about their acceptance and expectations for the new product and profit from their tacit knowledge to deduct requirements, ideas, and new potential use case scenarios (Bender and Gericke, 2021a). The goal is to create a space for the interaction between technology and society so that future developments can be actively shaped by and not only for the people involved (Šabanović, 2010). Glatte et al. (2019) showcase this by organizing workshops where they exhibit the designed prototypes in different contexts for a better understanding of the introduced technology. The participants get engaged in a discussion about their preferences and reluctances with the help of an object reflecting the product, a part, or function of the product. These discussions are moderated and will be documented for evaluation afterward. Showcasing prototypes to customers is also a tool called customer clinics used in the automotive industry to find out about user’s preferences, showing them new concept designs and retrieving their feedback (Pelka, 2018). Further tools for participatory design can be found in Brandt et al. (2013).

#### 3.2.3 Complemented activities

##### 3.2.3.1 Clarification of problem or task

We see the social and ecological dimensions underrepresented in the tools and methods for gathering requirements. Therefore, we want to provide a checklist for the ecological and social dimensions to be considered during this step of development. Since checklists are a common tool to help designers complete the list of requirements and not change the general approach, we figured this to be adequate and gathered directions to deduct possible requirements during our project and from different literature into one item.

**FIGURE 6:**
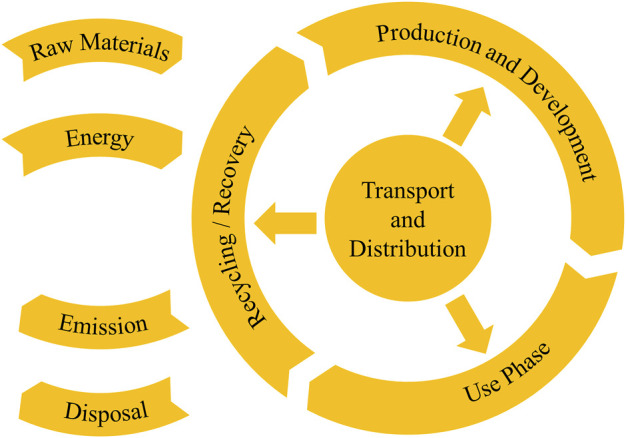
Product’s life cycle and inherent processes as described in (DIN EN ISO, 2021).

For the ecological dimension, we gathered our findings from McAloone and Pigosso (2021); DIN EN ISO, (2021); van Boeijen et al. (2020); Luttropp and Lagerstedt (2006); Wever and Vogtländer (2020) and structured them by the product life cycles and its inherent processes as depicted in Figure 6. The directions contained in the list, which are seen in Table 3, are meant to touch upon every aspect a product impacts the natural environment and remind designers to consider those. Clearly, not all of them can be fulfilled, as some interfere with each other or are not formulated as a measurable requirement but rather as a recommendation to improve.

**TABLE 3:** Checklist for ecological requirements.

Raw material extraction	Use phase	Recycling and recovery
⁃ Minimizing overall use of materials (volume per material, variety of materials)	⁃ Strengthen product-user relation	⁃ Use of recyclable materials
⁃ No use of toxic and dangerous materials	⁃ Minimize the use of consumables during the use phase	⁃ Include retrieval as a service (for recycling, remanufacturing, refurbishing)
⁃ Use of renewable and biocompatible materials	⁃ Reasonable lifespan of modules	⁃ Design to be easily cleaned and dismantled
⁃ Use of recycled materials	⁃ Increase the longevity of the product	⁃ Use of easy-to-dismantle connection techniques
⁃ Use of low-energy content materials	⁃ Increase the reliability of the product	⁃ Use of less, simpler, non-mixed materials and alloys
Production and Development	⁃ Design for upgrade and adaption possibilities	⁃ Modularity
⁃ Minimize production steps	⁃ Facilitate maintenance and repair by design	⁃ Design to be
⁃ Minimize production waste	⁃ Include maintenance and repair as a service	⁃ Reused (second-hand market)
⁃ Design as few connection components as possible	⁃ Design and manufacture to protect from dirt, corrosion, and deterioration or wear out	⁃ Repaired (life extension by fixing)
⁃ Minimize material consumption during design (virtual prototyping)	Energy Use	⁃ Refurbished (life extension by restoring quality)
⁃ Use of existing production sites	⁃ Choose non-toxic and non-dangerous sources of energy	⁃ Remanufactured (part of the product is remade)
⁃ Use of efficient production sites and methods	⁃ Choose renewable and biocompatible sources of energy	⁃ Retrieved (part of product is used in other product)
⁃ Minimize consumables during the production process	⁃ Minimize energy consumption during resource extraction and production	⁃ Recycled (materials are separated and upcycled or downcycled for other products)
⁃ No use of toxic materials during the production process	⁃ Minimize energy consumption during the development phase	Disposal
Emission to Air, Water, and Soil	⁃ Optimize energy efficiency during the use phase	⁃ Enable easy dismantling and sorting of materials
⁃ Reduce GHG emission (CO2, CH4, N2O, fluorinated gases)	Transport and Distribution	⁃ Collect gas and liquids during the use phase for extraction
⁃ Reduce the emission of volatile organic compounds (VOC)	⁃ Reduce transport volume	⁃ Minimize toxic byproducts and use closed circuits for them
⁃ Reduce the emission of acid potential gases	⁃ Reduce transport distance	⁃ Minimize portion of inevitable waste in product
⁃ Reduce the emission of eutrophic materials	⁃ Use of energy-efficient means of transport	⁃ Enable composting of materials
	⁃ Reduce or avoid packaging	⁃ Enable safe incineration
		⁃ Design and plan for milling

Social aspects always affect the circumstances of a group or an individual, e.g., a worker or a local community. The S-LCA focuses on activities during raw material extraction, production, distribution, and end-of-life, involving different groups of stakeholders (UNEP, 2020). Looking at mechatronic products and especially autonomous technology, the use phase becomes more complex and thus more relevant in terms of social impacts. To emphasize this novelty, we differ between “activities for the product” (all life cycles except the use phase) and “activities with/by the product” (use phase). After that, we follow the distinction of the S-LCA and group our directions by the stakeholder groups. Table 4 represents the list that is made up of the impact subcategories described in UNEP (2020) and our findings.

**TABLE 4:** Checklist for social requirements.

Activities for the product		Activities by/with the product	
Worker	Society	Worker	Society
⁃ Enable freedom of association and collective bargaining	⁃ Support public commitments to sustainability issues	⁃ No loss of employment	⁃ Comply with the General Data Protection Regulation (GDPR.eu)
⁃ No child labor	⁃ Contribute to equal and stable economic development	⁃ Consider cooperation/supportive activities instead of replacement	⁃ Avoid bias in AI
⁃ Fair salary	⁃ Prevent and mitigate armed conflicts	⁃ Consider/plan/create alternatives for workers in case of replacement	⁃ Design appearance, movement, and behavior for increased acceptance
⁃ Fair working hours	⁃ Support conscious technology development	⁃ Avoid physical straining/dangerous tasks	⁃ Design appearance, movement, and behavior to avoid stress, anxiety, violence, or frustration
⁃ No forced labor	⁃ No corruption	⁃ No creation of monotonous/mentally straining tasks	⁃ Consider visual and audial communicative devices to indicate intentions
⁃ Equal opportunities/no discrimination	⁃ Support the ethical treatment of animals	⁃ Consider operation time and shifting conditions for night and day	⁃ Match overall interaction with the public to service/task
⁃ Improve health and safety	⁃ Increase poverty alleviation	⁃ No reduction of task quality or position loss	⁃ Support transparency of purpose and technicalities
⁃ Improve social benefits/social security	⁃ Consider children’s concerns regarding marketing practices	⁃ Avoid polarization of qualification levels	⁃ Reduce the emission of noise
⁃ Promote equal and stable employment relationship	Local Community	⁃ Increase equal opportunity and accessibility	⁃ Increase health and safety
⁃ No sexual harassment	⁃ Secure access to material resources	⁃ Increase health and safety	⁃ Consider the impacts of failure during operation in public
Value Chain Actors (excluding consumers)	⁃ Secure access to immaterial resources	⁃ Design interaction to avoid stress, anxiety, violence, or frustration	⁃ Introduce test phases
⁃ Secure fair competition	⁃ Avoid delocalization and migration	⁃ Plan for/consider impacts of failure during operation	⁃ Consider operation time and shifting conditions for night and day
⁃ Promote social responsibility	⁃ Protect cultural heritage	⁃ Support transparency	⁃ Consider the time and location of the operation site
⁃ Support equal and stable supplier relationships	⁃ Secure safe and healthy living conditions	Consumer	⁃ No creation of hostile environments (noise, claimed space, appearance, movement, behavior, visual/audial effects)
⁃ Respect intellectual property rights	⁃ Promote respect for indigenous rights	⁃ Increase health and safety	⁃ No replacement of socially valuable contacts/services
⁃ Support wealth distribution	⁃ Support community engagement	⁃ Design feedback mechanism	⁃ Increase equal opportunity
	⁃ Support local employment	⁃ Secure consumer privacy	⁃ Increase accessibility to public spaces or services
	⁃ Enable secure living conditions	⁃ Support transparency	⁃ Support anthropocentric implementation
	⁃ Support education in the local community	⁃ Take end-of-life responsibility	⁃ Reduce conflicts of interest for public space
		⁃ Avoid health issues for children as consumers	

Determining which requirements will be mandatory, what the thresholds are, and which to put aside for remaining capacities has a big influence on the evaluation and assessment of each developed solution concept. Since mandatory requirements are the criteria for excluding concepts, they will not be part of the assessment itself. The remaining requirements will decide on the quality rating and final choosing of a solution concept. Herein lies the second conflict of interest we identified, as the fixing of requirements is already a process that dictates the direction and the product’s performance in each dimension of sustainability. Making decisions here has to be discussed and supported by the whole team and approved by the person responsible.

##### 3.2.3.2 Definition and weighting of assessment criteria

This activity is well documented and follows a generic procedure described in Wartzack (2021). We do not suggest changes to the general approach but complement the ecological and social dimensions. As motivated before, we split the original activity in the VDI2221 into two parts. For us, the first step lies in the definition and weighting of the criteria so that this is described by the following actions.

1. The deduction of assessment criteria from requirements.
2. The definition of a scale.
3. The weighting of the criteria.
4. The documentation and illustration of results.

The first and the last bullet point will not be explained any further since we do not offer any addition to those. In order to assemble assessment criteria, we recommend following the guiding rules described in Wartzack (2021).

Scaling can be described as the transformation of qualitative and quantitative criteria, making them comparable and unitless. In this process, there will always be a loss of information and the issue of subjectivity (Verein Deutscher Ingenieure, 1998). Other challenges arise from the comparison of different units of quantitative criteria (e.g., emission of greenhouse gases (GHG) and usage of water) and the scaling of qualitative criteria (e.g., acceptance of society and quality of work due to automation). The VDI2225 describes the same issues for functional assessment criteria and introduces a scaling from zero to four by which both quantitative measures with different units and qualitative aspects should be evaluated. The values are expressed as 4 = very good, 3 = good, 2 = fair/pass, 1 = bearable, and 0 = unsatisfactory (Verein Deutscher Ingenieure, 1998). In the same way, social studies try to quantify qualitative aspects in surveys with the help of the Likert scale where usually five levels of agreement are given to rate a statement connected to the aspect (Bryman, 2012). However, this has to be done by an interdisciplinary team with the help of experts in the different categories of sustainability to minimize the mentioned issue of information loss and subjectivity (Wartzack, 2021). Additionally, the general approach of scaling and trade-off during evaluation entails other problems that will be discussed in Section 5.

For the weighting, we follow the pattern of the value-benefit analyses by Zangemeister (2014). The goal is to structure all assessment criteria in a hierarchy for each dimension and assign weights. We suggest keeping the ecological, social, economic, and functional dimensions apart and not reducing them to one value. This is already recommended for the functional and economic dimension in the VDI2225, as they describe a single value to be less descriptive and meaningful, mostly because it will not indicate where the strengths and weaknesses of the evaluated concept lie.

While the weighting of the criteria determines the outcome and decision for a solution concept, it is important to note that this represents the third conflict of interest we identified during our project. The distribution of weights reflects the priorities, values, and strategical direction of the company and the designers. As it will always be a compromise, the results need to be discussed and supported by the whole team and approved by the person responsible.

##### 3.2.3.3 Assessment and selection of solution principles

Gathering necessary information for the evaluation of the ecological and social dimensions increases the time spent on this activity significantly. Depending on the level of detail, it requires virtual and physical prototyping, simulation, and data acquisition for life cycle assessments in three dimensions, surveys, interviews, and workshops. As before, we focus on the ecological and social dimensions. For further input on the functional and economic aspects, we recommend reviewing Bender and Gericke (2021b) and 2225 Blatt 3:1998-11.

For the ecological aspects, the main evaluation tool is the LCA. The goal of the designers during this procedure is to estimate the material composition in the product as well as the energy consumption and related material flows during the use phase. The material flow analysis (MFA) describes the systematic approach to find and document all in- and outputs for a product over its life cycles (Brunner and Rechberger, 2004). This requires a certain maturity of the concepts with existing virtual prototypes and an idea of used components and outsourced items. For an estimation of the energy consumption during its use phase, we suggest integrating the functional units in a simulation that represents the real-world application of the product. This is especially important since research shows the significance of this phase regarding GHG emissions ranging from 70% to well over 90% (Wyatt et al., 2017; Li et al., 2021). Bergerson et al. (2020) recommend simplified LCA or screening approaches for this stage to help identify strengths and weaknesses in the concept and allow focusing on these for the assessment and rating of solution concepts.

In the social dimension, we recommend the procedure of the S-LCA in combination with the guidelines for automation initiatives. The process of the S-LCA resembles that of the LCA and is well documented in UNEP (2020). For the guideline, the gathering of information is implemented with the help of interviews, surveys, and workshops with the stakeholders involved. This is similar to the last step of the PSA but with a focus on evaluation rather than openly discussing concept options. The challenge herein lies in the processes going beyond the usual workflow of a design team, reaching into social science disciplines. Depending on the level of detail and allocated resources for this topic during development, experts should be consulted for the validity of the results.

The actual rating of the concepts should be a product of discussion and compromise of a diverse group in the same way as the scaling process. It is important to be aware that even quantitative criteria will be influenced by subjects and hence be subjective. All results, especially concepts that are close in their rating, should be critically questioned and undergo a closer examination (2221:2019-11). Therefore, Wartzack (2021) suggests the weak point analysis of concepts and a sensitivity analysis of assessment criteria.

## 4 Case study

The methods we suggest for the integration of the ecological and social dimensions described in Section 3 were gathered or developed during the project MARBLE (Mobile Autonomous RoBot for Litter Emptying). This chapter introduces the case study and presents the results of the PSA and the evaluation of the ecological and economic dimensions of our first concept.

### 4.1 Project MARBLE

In the city of Berlin, Germany, the municipal waste management (BSR) maintains the public litter bins in the streets and parks, resulting in around 6.2 million emptying processes per year. The fleet dedicated to that task consists mostly of gasoline-fueled cars (two out of 49 were electrical). The project MARBLE by TU Berlin set out to optimize energy consumption and avoid CO2 and local NOx emissions. Therefore, we investigated how far a specially developed service robot can support and improve the process of emptying the public litter bins. The goal was to build a prototype to verify the technical feasibility and include all dimensions of sustainability in the development. The Project set out to focus on the ecological dimension with the condition not to disrupt the labor force while estimating the cost of such an endeavor. As the focus changed during the course of the project, we emphasized the process around the social dimension with a detailed description in the following section. Since the PSA evolved and was not ready to use from the beginning, we illustrated the steps in chronological order, which might deviate from the formalized steps that we discussed in section 3.

### 4.2 Preliminary social assessment

**FIGURE 7:**
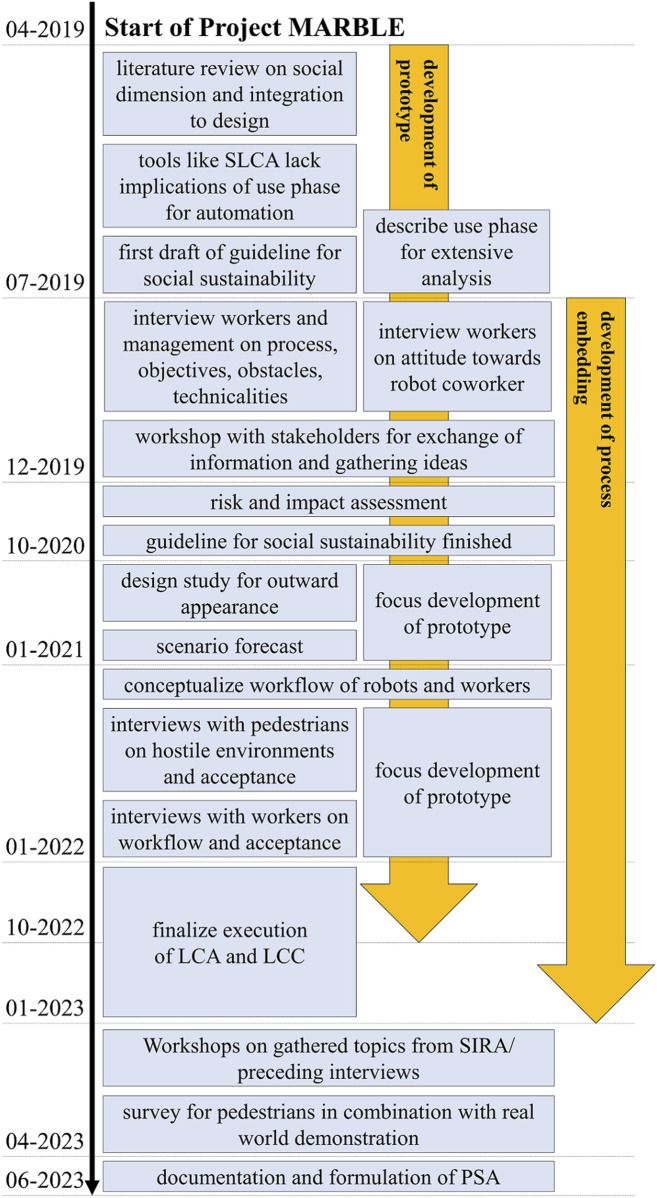
Timeline of steps for the social dimension along the development of MARBLE.

For an overview, Figure 7 shows a timeline of the project and the steps taken to investigate the social impacts of MARBLE. The initial concept idea for MARBLE was to operate on the pedestrian path and empty bins autonomously. This job was perceived to be done reluctantly and we aimed to free capacity for other tasks within the BSR as they struggle with personnel. So, the first activity was to investigate the basic conditions, the mechanical procedure, and the people involved. Therefore, we accompanied the BSR workers on their tour interviewing them on conditions and occurrent complications to get an overview of the operation sites. The main issues arising were the accessibility due to blocking obstacles or limited space. We also found that depending on the time of day and location, the amount and frequency of pedestrians vary greatly, which could influence the maneuverability and workflow. Also, the robot has to move on the sidewalk, limiting its size and speed. Workers usually empty around 200 bins during one shift and store the garbage in their trucks which are equipped with a compacter. For a small-sized robot, this requires some form of depot in close proximity to dump the collected garbage or a bigger-sized truck (mothership) that empties the robots. Additionally, the robots run on electrical energy to avoid local emissions, making it necessary to charge or change the battery. When asked about introducing a robot to their workflow, they seemed untouched and skeptical about the technical feasibility.

In the process of gathering requirements, it became clear that tools for social sustainability were not easily available within product development processes. Tools like the S-LCA or studies on the acceptance of SARs did not satisfyingly cover our case so we started drafting a first guideline tailored to the introduction of autonomous systems. While creating the guideline, we realized the necessity for an extensive description of the use phase and the embedding of the product into the process. This led to a first workshop, where we presented our drafts to the BSR (members from different departments) to exchange ideas and openly discuss concerns, obstacles, and improvements.

Since the service robot operates in public, it will move alongside pedestrians, including children, people with visual impairment or mobility aids, cyclists, or pets which need to be considered. There will also be forms of interaction with the BSR workers or technicians regarding control, battery change, garbage removal, service, or maintenance. Taking these stakeholders into account, we especially had to consider the time and place of operations. For example, operation during the night will entail the problem of noise disturbance and require the BSR to introduce nightshifts, as long as the system is not completely autonomous. Another big concern was vandalism and how to deal with it. Reflecting on such issues from different perspectives helped further the guideline and the second step we later called SIRA.

The first SIRA was conducted with a group of developers from different disciplines (mechanical engineering, environmental engineering, philosophy, business administration, and Engineering). Here, the fear of changing the job to a more monotonous position and the creation of hostile environments with unclear responsibilities was predominant. It was also noted that the implementation needs to be carefully planned to not replace workers but support them without changing qualification levels too much. As a benefit, the robot could alleviate physically straining tasks and increase job accessibility for people with disabilities. As a result, we wanted to include the stakeholders more in the development of the robot and embed them into the process, as our vision changed from autonomous operation to a concept of cooperation. The occurring interactions with people in public made research the topic of design concerning behavior, movement, and appearance. Human-like motions, audiovisual signals, and cute or human-like looks can increase acceptance and decrease fear or vandalism.

In the next step, we wanted to validate and analyze our assessment and first conclusions. A literature review on this topic confirmed the experience of little engagement during the very first interviews and early workshops. With the limited time frame of the project and one goal being the verification of the feasibility, we now concentrated on the development of a functional prototype to use for the step we later called acceptance analysis and validation of needs. For the same matter, a product designer helped create a design study implementing cute and human-like features. In the scenario building, we included the results from the SIRA and conceptualized two possible operation modes: the mothership scenario and the service station scenario. In both scenarios, a fleet of USRs supports the BSR in emptying litter bins. The first is complemented by the mothership, which is an electric vehicle, operated by a human to empty the robots and change empty batteries. Service stations will be spread around the city in the second scenario, where the robots can empty their compartment and recharge. Following the scenario technique, we extrapolated trends and assumptions to complete a picture for our USR in the near future. It consists of a growing city population and an increase in tourism which could result in a growing quantity of waste. The structural changes in the city could lead to more space for pedestrians and less space for private transport. The creation of service stations would benefit from this, but motherships could have difficulties reaching the service robots when streets get closed completely. However, operating a robot on pedestrian paths along with service stations demands space so a conflict could arise between pedestrians and robot operators. The advancement of technology might have a positive influence on the general level of acceptance of autonomous robots in public and reduce the danger of vandalism. With autonomous driving introduced, the legal framework already exists and autonomous USRs will easily conform.

**FIGURE 8:**
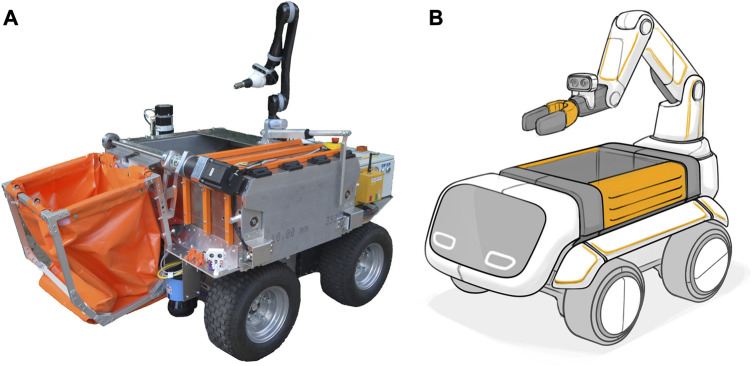
Functional prototype **(A)** and conceptual design **(B)** in the project MARBLE.

With the design study (Figure 8B) and the first functions to demonstrate, we interviewed workers from the BSR and different groups of pedestrians (with mobility aids and visual impairment, as well as without a home). The interviewed pedestrians and workers underlined possible arising conflicts in high frequented areas during rush hour and again raised concerns of vandalism as inevitable. The general acceptance in their view is influenced by the efficiency and the outer design, which needs to be clear about the purpose of the robot. They both think it a good idea to start with test phases in more confined areas with less traffic like city parks. They had no concerns about safety and trusted that a deployed robot would be well-licensed and verified. The pedestrians noted that increasing the service of street cleaning could actually benefit the wellbeing of the citizens since they criticize too many full and overflowing bins. Important for them was to consider minorities and adapt the design accordingly. Interestingly, the perception of emptying bins diverges between workers and pedestrians. The latter see it as degrading and inhumane while the interviewed workers appreciated their job. The workers also highlighted the importance of the human—robot interaction to be easy and not stressful. They do not trust the technical feasibility yet, as it seems to be slow and inefficient. As a consequence, they fear the additional responsibilities of taking care of the robot and thus less time to concentrate on their original job. However, stating that older or injured colleagues are limited in taking on physically straining tasks, they see the potential for benefits by introducing a service robot. For the last workshops and survey, we first finalized the functional prototype (see Figure 8A) to demonstrate the robot operating the bin. The workshops were held with BSR personnel (four to six per workshop) from different organizational units (street cleaners, machine operators, repair shop staff, and route planners). It was important for us to do separate workshops keeping the managing staff apart from the street workers in order to avoid hierarchical influence on the discussions and statements made. The workshop began with a short introduction and demonstration of the prototype. After that, a moderator led through several topics (functionality, challenges, safety, acceptance, and impacts) inciting and guiding the discussions with the help of prepared questions. The discussion and statements were documented afterward. In the next section, a brief summary of their views is described. In the same phase, a survey was distributed among pedestrians during an open demonstration of the prototype in the streets of Berlin and we received 1131 replies. This helped to get their view on the robot since we had a limited capacity of workshops and aimed for higher participation rates.

In general, the workers from the BSR were very open to the idea of USRs supporting them. They could imagine taking over new tasks like changing the battery, handling some hardware maintenance, or steering it when stuck. These had to be easy-to-learn tasks and linked to their original job; anything related to software issues was excluded. Since there are already places with litter bins restricted to cars, they see advantages in a small robot. These are mostly park areas or squares, which the BSR sees as a suited test scenario anyway. They do not trust the robot to be effective in crowded streets except for nightshifts. Another input from the workshop was to adapt the robot and use it for other tasks like winter service or mowing of weeds. Furthermore, the feedback confirmed the potential benefit of the robot for the aging workforce by taking over physical straining tasks, making some activities more accessible to them. They disproved the fear of creating monotonous work since most workers are used to it and even appreciate it, as long as it will not confine them to a desk. We also found that the workers were predominantly curious about new technology and they expressed a certain pride to be part of the modernization. However, technical complexity could overburden some and they are afraid to be responsible for an expensive robot. Also, introducing an autonomous robot generates the fear of being replaced. They suggested limiting the number of robots so not all workers are obliged to adapt to the new tasks and it will feel more like an extension of the fleet.

The survey consists of statements connected to social aspects that have five different levels of agreement to choose from (e.g., strongly agree, somewhat agree, neutral, somewhat disagree, and strongly disagree). The participants were mostly in the age group of 30 and 49 (58%), 27% were younger, and 15% were above 50 years old. Generally, the attitude toward service robots like the one from the project MARBLE was positive and accepting. Nearly all participants strongly or somewhat agree (42% and 50%) that a robot can support the BSR and would feel safe around it. Interestingly, 78% said that they would accept service robots in urban spaces as normal in around 1–5 years, but they think it will take at least 5–10 years for the necessary technological maturity (76%). Particularly, the aspect “safety and accident avoidance” was chosen as the feature needing more development (50%). The impact of service robots on the urban infrastructure was seen as positive (42%) and somewhat positive (47%). However, the majority strongly or somewhat agree (45% and 46%) that it would change the pedestrian’s behavior and movement, hence this should be further investigated and considered in the development. The need for social and environmental regulations on the introduction of robots to public spaces was also agreed upon by nearly all participants: 43% strongly agreed and 53% somewhat agreed. The conceptual design of the robot was perceived as visually appealing although from several options, the participants favored modern design and human features over a cute look. The statistical analyses of the survey resulted in finding strong correlations between acceptance and the topics of sustainability, job quality for the BSR, design, and safety.

The workshops and survey took place at the end of the project and are ready to serve as input for the next iteration of a service robot with municipal tasks. Within the project MARBLE, this is as far as we got along with formulating these experiences into the PSA, hoping it will serve as a practical example and useful method.

### 4.3 Life cycle assessment

In our first life cycle assessment, we compared our case study with the current fleet, consisting of 37 vehicles running on gas and 12 on electricity. The global warming potential (GWP) caused by the operation of this fleet impacted the whole outcome to clearly favor the use of our robot. However, since the BSR has committed to completely electrifying its fleet, we compare our two MARBLE scenarios with an electric fleet from the BSR in order to make this study valid for future comparisons. We also use the electric energy mix for the year 2030 based on the assumption of Agora Energiewende et al. (2022). The calculations for the results are based on the inventory analysis, meaning the gathering of data on all components and a simulation of the energy consumption. By measuring the energy consumption of the operations in the physical prototype, we validated the simulation data.

The inventory analysis is based on the prototype, presenting a first estimation. Gathering this data is cumbersome since most bought components, especially electronic devices lack information about the material composition and we could not afford to disassemble all parts for an exact identification. The used data for those parts rely on examples of similar components with partly size adjustments, making this another source of uncertainty. As mentioned before, the LCA in such an early stage will always come with uncertainties. It is therefore important to disclose these issues, as we might base decisions on the results. In the same manner, it is becoming common to use a 20% rule. It says that the difference between two scenarios, concepts, or products has to be above 20% to be deemed significant (Matthews et al., 2014).

**FIGURE 9:**
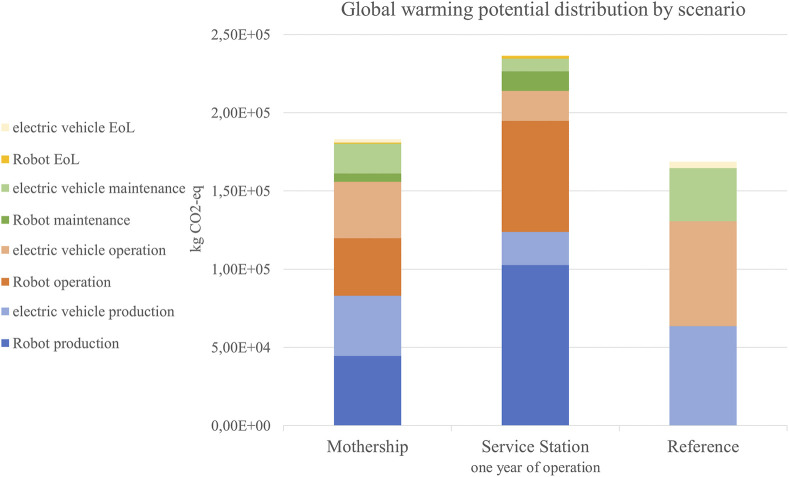
Scenario comparison of calculated global warming potential during a 1-year operation.

In the category of GWP, Figure 9 shows that there is no significant difference between the mothership scenario and the reference, while the service station scenario produces the most (+40%). This is due to the fact that charging the battery results in more downtime for the robots and thus more robots are required to do the work at the same time. They also drive more distance to reach the service stations. The biggest impact is caused by the energy consumption (30%) during operation and the production (43%) due to the use of aluminum, copper, and rare-earth elements. In the mothership scenario, fewer robots are calculated by the simulation, hence the impact of production is 24% and consumption is 20%. The motherships’ production and operation take up around 20% each. The relatively low contribution of the use phase to the overall GWP (around 60%) is due to the energy mix calculated for 2030 with a high portion of renewable energy.

Next to the GWP, the LCA also looks at aspects like water consumption, land use, or terrestrial ecotoxicity (ReCiPe midpoints impact categories). Here the main drivers are the production of batteries and electric motors as well as the materials used for constructing the robots or electric vehicles due to the extraction processes. The mining and refining of, e.g., aluminum, copper, nickel, zinc, lithium, or chromium causes pollution and damage to human health by toxic substances used, fresh water shortage, and the destruction of landscapes and ecosystems. These impacts can be summarized in the endpoint indicators. While the results for the service station scenario remain the worst of all three scenarios, the mothership scenario was just slightly worse than the reference in damage to ecosystems (7%) and to resource availability (4%) but 5% better in damage to human health. Therefore, we can state that there is no significant difference based on the current data.

### 4.4 Life cycle costing

For the life cycle costing of our prototype, we included an estimation of the costs for software development, purchased parts, production of the robots, maintenance, and repair during the use phase. To put this into a reference for a conclusion, we took the number of emptying processes and calculated the current costs from the data given by the BSR. This comparison will only reflect the monetary costs of the service and neither touch upon any social-economic aspects nor suggest the replacement of human workers.

The current process by the BSR results in around 8 million € per year with the labor costs taking up 81% of the share. For a fleet of robots, the costs add up to around 6.3 million € per year. However, this estimation should be treated carefully. Especially, the use phase is unprecedented and, therefore, uncertain in its results. The personnel needed to maintain robot operation or the loss caused by vandalism cannot be put into valid numbers yet. Still, the LCC provides us with information on the distribution and most impactful factors. For example, the prototype has a robot arm to handle the complex motions to open the litter bin. Adapting the bins to robot operation and replacing the arm with a much simpler device yields an improvement of 20%. Another cost driver is the battery. Improving the capacity results in fewer robots needed and also less batteries used up per robot, resulting in an improvement of 15%.

## 5 Discussion

### 5.1 Gathering data for assessment

We learned that it is very helpful for the PSA to have physical prototypes with a functional display of the tasks to engage the workshop participants. Although speculative design also helps to engage the participants, we found that in earlier encounters with no functional prototype, skepticism toward the technical feasibility was high. We observed that the statements made by participants got more concrete as they were facing the functional prototype. We assume that this is connected to the innovative nature of a USR, as it lacks similar existing products to be put in relation to.

Also, the gathering of feedback and comments by stakeholders is only the first step. Taking the information into account and translating it into design choices is a complex task and susceptible to misunderstanding. Therefore, the participation has to go on and complement the process. The results and conclusions have to be communicated transparently in a way that participants will be able to see their efforts put into action, as their suggestions might be neglected. The genuine intent to use tools for engaging stakeholders in the development process requires thorough planning and evaluation, as there are limitations and dynamics to be considered (e.g., stakeholder representation, appropriate mode of participation, execution, and documentation) (Felt and Fochler, 2010).

For the LCA and LCC, a basic concept of the robot and an understanding of its embedding within the whole process was necessary. Incorporating these evaluation methods, especially the PSA, will take up a lot of time and financial resources, which is an obvious drawback. The benefit lies in the documentation of the results and that they can be reused in modified and redesigned versions of the product. As our service robot is based on a modular design, the assessment of each module can be transferred or adapted. Building up the product structure early may result in extra work due to changes in the concept but will also help in the overall process of structured development. It remains questionable whether companies can afford this additional time and cost in the development of their products. However, we argue that this is necessary to arrive at a concept that considers all dimensions of sustainability and actually improves the result.

The data we gathered for the inventory analysis for the LCA can serve as the basic input for a complete S-LCA, which we did not conduct due to time and resource constraints. We rather focused on the use phase as this seems unprecedented. However, we learned from the LCA that the most impactful materials are almost equally used in the reference and robot scenarios. So, we can assume that the difference in those scenarios concerning the impacts discovered in the S-LCA will not be significant.

### 5.2 Project results

Within the scope of our project, we did not come to decide on a final solution concept. Particularly, the assessment of the ecological dimension showed that introducing a service robot yields no significant improvement compared to electrifying the entire fleet. One of the challenges is that some requirements connected to the ecological impact often counteract functionality issues or even ecological aspects themselves. For example, the choice of material is mainly bound to specific parameters to ensure stability and longevity of the construction and withstand occurring forces and weather conditions. Steel and aluminum fulfill these requirements but contribute to the GWP and a lot of the midpoint indicators in the LCA. In the same way, electric drives and the battery are made of resources that have a heavy influence on the ecological dimension. One way of improvement will be the reduction of weight. This will result in less material used and less energy consumed during operation. The motor and batteries have to be managed carefully and optimized for longevity. For instance, using the battery’s state of charge (SoC) between 10–70% increases the possible number of recharge cycles drastically in comparison to using the full range of SoC from 0–100%. Also, a redesign might help to reduce the number of drives and sensors needed, as we successfully prototyped a new way to operate the bins, eliminating the robot arm. More of these small improvements could refine the concept to score much better in the LCA and actually present a more ecologically sustainable alternative.

These suggested improvements are directly related to the economic aspects we evaluated. Fewer robots, less energy consumed, fewer materials required, and fewer electrical drives or sensors used directly lowers the cost of operation and therefore improves profitability. Going by the extensive definition of economic sustainability we supplied in Section 2, the robot should also improve the quality of life and services. As interviewed pedestrians criticized a lack of service regarding waste collection in the public space and the discomfort caused, our concept has the potential to improve service and quality of life by adding to the workforce. Of course, another way to solve this problem resides in decreasing waste generation. Although our scenario analyses deemed this outcome improbable, it is a more desirable solution to be discussed in another scope.

In terms of the social dimension, our concept changed throughout the process. We learned that the general idea of a robot working in an urban environment seems quite accepted under a few conditions. As replacement is the biggest fear of the employees, we need to make sure to find the right application that is either not taken, not feasible by humans, or unpopular. On the other hand, pedestrians mostly fear the conflict of space distribution so for the development of any USR, it is necessary to consider the time and location of the operation as well as incorporate possible structural changes in the city. In any case, the development process should be transparent and inclusive regarding the stakeholders. After the workshops, the participants expressed their appreciation and that the acceptance of such an innovation increases with each participation in the process.

### 5.3 Evaluation and decision-making

Another topic is the comparability of the results and the scaling of assessment criteria. This holds especially true for the ecological and social aspects. The LCA results in quantitative indicators which makes them measurable and easy to compare to other scenarios. The problem here lies in the actual understanding of these indicators, as they are hard to grasp and intangible. Also, the individual indicators are hard to compare to each other and therefore hard to balance as they are measured in different units. Nonetheless, this method supplies the designer with information that can be used to reduce the impact. Clearly, we could identify the most impactful components or processes and tend to minimize them. In that regard, the method succeeds in considering the environmental dimension. As every product will impact the environment in one way or another, the question is: How much is acceptable and when should we as designers decide to discard a concept completely? However, how to go about the results is not within the scope of this method and probably needs to be determined on a political level.

The lack of standardized methods to incorporate the social dimension made us focus on this aspect the most during our project. The suggested PSA that we applied yielded many results and gave us an insight into possible impacts such a service robot might have. We are sure that it is impossible to foresee every impact on and reaction by society, but the amount of input we received by this method that otherwise would have been neglected shows how important and valuable this method is. The challenge inherent to the social dimension is that a lot of indicators are qualitative values that can neither be measured, expressed in units, nor compared to other criteria. We mentioned this in Section 3 and suggested a tool, which is used for the unitless comparison of functional and financial criteria.

However, this procedure cannot solve the fundamental problem of trade-off and the questionable transformation to match one unified scale. This is because the assumption to express all criteria in commensurable values implies the idea that the loss in one criterion can be compensated by increasing another (van de Poel, 2020). This might work for economic aspects but not for moral values (equality, quality of work, etc.) or criteria describing the pollution of freshwater or global warming. With our approach, we cannot offer a solution but want to underline the importance of being aware of this problem/dilemma. The first step to improve the assessment regarding this problem will be to look closely at the evaluation and not only the scoring since its representation should be questioned. Scoring from zero to four as the VDI2225 proposes can be based on the comparison to another product or scenario (dependent on ambition) or one’s understanding of social and ecological sustainability. As we said before, this is influenced by the corporate strategy and values of the company and the designers. The decision-making needs to be based on an interdisciplinary team that discusses the pros and cons. Our tool shall empower the ecological or social expert to have data and information to base their standpoint on and argue for or against a concept. The requisite is that there is such a team composition and that people in charge account for each dimension equally.

### 5.4 Validity

In Section 4, we already mentioned the problem of validity in such an early stage of the concept. On the one hand, flaws in the application or calculation can occur, and on the other hand, the aspect of uncertainty is inherent to the early use of such assessment tools. By updating the LCA and LCC twice during the project, we eliminated most errors. The uncertainties could not be eliminated but became clearer through the updates and can be categorized into three topics: The material composition, the energy consumption simulation, and the early stage of the concept. The first one refers to the lack of data concerning the material composition of purchased parts. This led us to assume the composition by taking similar products we could find in the database, resulting in uncertainties. The second is based on a virtual prototype and a virtual process simulation that cannot perfectly reflect reality and hence introduces another source of uncertainty. The last one expresses the uncertainty of the whole concept not being finalized. Components or modules might change and the embedding of the robot is not yet at a final stage. With this in mind, we once more point to the 20% rule (see sect. 4.3), yielding no significant difference in the ecological dimension but a significant improvement concerning the cost of the operation.

### 5.5 Transferability

The LCC, LCA, and S-LCA are tools made for any product. The checklists we presented also originate mostly from existing tools intended for any sort of product. Additionally, checklists help to complete the list of requirements and are by no means mandatory, making them suitable for any product. Only the PSA has a special focus on the use phase and was developed along our case study of a USR. We see many use cases for municipal services that can be offered by such products. We see this method fit for such applications. On a higher level of abstraction, MARBLE is an automated mechatronic product. It is able to replace or supplement human workers as other machines already do in factories or might in the future of some service sectors. The PSA could in that case result in a different target group but still be useful to capture the requirements of those stakeholders. For products that do not perturbate public space or working conditions in the same manner, we believe it to be a too costly effort.

## 6 Conclusion

The goal of our method is to be applicable in the design process of service robots and other complex automated mechatronic products. To include both the ecological and social dimensions, the designers are required to engage with new topics and disciplines. Naturally, this will depend on the qualification of the designers and the composition of the design team, hence external expertise might be required. As companies are starting to introduce environmental management and engage with corporate social responsibility and responsible research and innovation, we also see a change in education throughout technical sciences (Kattwinkel et al., 2021). This can help further the application of methods considering all dimensions of sustainability, but we still see a challenge in the motivation to do so. We did not set out to create incentives but to deliver a tool that offers designers help in case sustainability is prioritized.

As we described above, those methods to assess the ecological, social, and economic impacts exist. In our case, we developed a guideline and the PSA for the social dimension of the use phase, as this special case of USRs has not been satisfactorily covered. But for the most part, we could resort to existing methods and gather the information in the checklists we presented. We believe these tools to be a good supplement, integrating easily into the existing PDPs. The implication of the process is foremost an increase in time and cost of the development, resulting in possible challenges for the implementation due to a lack of resources (Bertoni, 2017; Wever and Vogtländer, 2020). It will be necessary for companies to communicate the value creation opportunities by implementing those measures thereby making it attractive for investors (Bertoni, 2017; Rojas and Tuomi, 2022). Not only the application of the tools but also the resulting conflicts and necessary trade-offs will require new expertise and a paradigm change in order to unfold their potential. As mentioned before, this constitutes more than just new development tools but, for example, change in the energy sector and on a political and societal level that is clearly out of scope for this paper.

The case study presented in this paper delivered insightful results on all dimensions. We learned that the scenario and related processes play a huge role so that we cannot only look at the robot or product itself but also how it is embedded into the service and what else is affected. Obviously, in our case, the number of robots is one of the biggest factors for the economic and ecological dimension so the robot needs to be more efficient and the process optimized to require fewer robots in the operation. From the functional point of view, it seems difficult to deal with the complex operation with the current infrastructure. In that regard, we learned during the workshops that there might be other tasks easier to automate and with less complex parts that are more accepted to be taken over by a robot. The PSA revealed that a lot of the workers are content with their tasks despite them being monotonous. They expressed the fear of being replaced and wanted to make sure that they had a say in where a robot would be deployed. Service robots in general seem accepted and part of an inevitable future to the workers as well as the denizens of the city. The analyses also showed its relevance and how such an undertaking can profit from the participation of stakeholders.

For the prototype developed in our project, we saw mostly positive impacts in the social and economic aspects, while the ecological dimension yielded no significant improvement. The concept is thought to be modular in a way that the platform can serve as a base for interchangeable modules delivering a variety of services for the municipality. For the case study, we settled for the emptying of litter bins, with other use cases in mind. At the current state, it seems that other use cases generated in the workshops might be easier to implement, as the current state of litter bins in the city of Berlin poses a complex task. The results continue to be useful for further development in that direction.

With this paper, we hope to contribute to research and support efforts to understand the social implications of technology better and make the integration of sustainability aspects in the design process more applicable. Our findings are yet to be used in more practical applications in order to be reviewed and refined as we see them as a work in progress in a challenging topic.

## Data Availability

The raw data supporting the conclusion of this article will be made available by the authors, without undue reservation.
